# Evaluation of three protocols for direct susceptibility testing for Gram-negative rods from flagged positive blood culture bottles

**DOI:** 10.1128/spectrum.03081-23

**Published:** 2024-03-06

**Authors:** Salman Khan, Arghya Das, Anwita Mishra, Ashima Jain Vidyarthi, Mukesh Nandal, Himanshu Yadav, Shayak Roy, Mahipal Singh

**Affiliations:** 1 Department of Microbiology, National Cancer Institute, All India Institute of Medical Sciences (Jhajjar-campus), New Delhi, India; 2 Department of Microbiology, All India Institute of Medical Sciences, Madurai, India; 3 Department of Microbiology, Mahamana Pandit Madan Mohan Malviya Cancer Centre and Homi Bhabha Cancer Hospital, Varanasi, India; 4 Department of Emergency Medicine, National Cancer Institute, All India Institute of Medical Sciences (Jhajjar-campus), New Delhi, India; 5 Department of Oncoanaesthesia and Palliative Medicine, National Cancer Institute, All India Institute of Medical Sciences (Jhajjar-campus), New Delhi, India; NHLS Tygerberg/Stellenbosch University, Cape Town, Western Cape, South Africa

**Keywords:** direct, antimicrobial, susceptibility, blood culture, categorical agreement, disk diffusion

## Abstract

Bloodstream infections are associated with high mortality, which can be reduced by targeted antibiotic therapy in the early stages of infection. Direct antibiotic susceptibility testing (AST) from flagged positive blood cultures may facilitate the administration of early effective antimicrobials much before the routine AST. This study aimed to evaluate three different direct AST protocols for Gram-negative rods from flagged positive blood culture broths. Blood culture broths showing Gram-negative rods only were subjected to direct AST by Clinical and Laboratory Standards Institute-recommended direct disk diffusion (protocol A). Additionally, automated AST (protocol B) and Kirby-Bauer disk diffusion (protocol C) were performed with standard inoculum prepared from bacterial pellets obtained by centrifuging blood culture broths in serum separator vials. For comparison, conventional AST of isolates from solid media subculture was also performed with Kirby-Bauer disk diffusion (reference standard) and the automated method. Overall, categorical agreements of protocols A, B, and C were 97.6%, 95.7%, and 95.9%, respectively. Among Enterobacterales, minor error, major error, and very major error rates of protocol B were 3.5%, 0.36%, and 0.43%, respectively, whereas minor error, major error, and very major error rates of protocol C were 3.4%, 0.72%, and 0.21%, respectively, and among non-fermenters, protocol B had a minor error rate of 6.5%, and protocol C had a minor error rate of 4.1% and major error rate of 1.9%. All three direct AST protocols demonstrated excellent categorical agreements with the reference method. Performance of protocols B and C between Enterobacterales and non-fermenters was not statistically different.

**IMPORTANCE:**

Bloodstream infections are associated with high mortality that can be reduced by targeted antibiotic therapy in the early stages of infection. Direct antibiotic susceptibility testing (AST) from flagged positive blood cultures may facilitate the administration of early effective antimicrobials much before the routine AST. Clinical and Laboratory Standards Institute-recommended direct AST can be performed with a limited number of antibiotic disks only. On the other hand, using an automated system for direct AST will not only allow effective laboratory workflow with reduced turnaround time but also provide the minimum inhibitory concentration values of tested antibiotics. However, using expensive automated systems for direct AST may not be feasible for resource-limited laboratories. Therefore, in this study, we aimed to evaluate the CLSI-recommended method and two other direct AST protocols (one with an automated system and the other with disk diffusion) for Gram-negative rods from flagged positive blood cultures.

## INTRODUCTION

Bloodstream infections have been associated with high morbidity and mortality rates. The crude mortality rate is estimated at around 15%–30% (1). Timely administration of appropriate antibiotics is crucial since a 9% increase in the odds of mortality might occur with every elapsed hour of delay in the antimicrobial administration of a sepsis patient (2). To prescribe appropriate antibiotics to patients with bloodstream infections, clinicians rely on routine microbial testing, which involves the following steps: blood culture in broth, subculture on solid medium, microbial identification, and antimicrobial susceptibility testing (AST), with a turnaround time of 36–48 hours (3). Hence, they are compelled to prescribe broad-spectrum antibiotics empirically until routine identification and AST reports are available. However, a very high percentage of antimicrobials prescribed empirically is found to be inappropriate, leading to adverse outcomes in the initial stages of the infection (4, 5). The other inevitable consequences are the emergence of antimicrobial resistance and the substantial increase in healthcare costs (6, 7).

Therefore, the possibility of performing AST directly from flagged positive blood culture bottles has been explored by researchers and has been shown to decrease the length of hospital stay, reduce healthcare costs, and modify antimicrobial therapy in clinical outcome-based studies (8). Various techniques have been developed for that purpose, from simple disk diffusion to AST on chromogenic agar and automated AST to Raman microspectroscopy (9–11). Recently, the Clinical and Laboratory Standards Institute (CLSI) and the European Committee on Antimicrobial Susceptibility Testing (EUCAST) published breakpoints for rapid disk diffusion methods directly from flagged positive blood culture broths (12, 13). Although both CLSI and EUCAST disk diffusion methods are useful for resource-limited settings, these are constrained with breakpoints of limited antimicrobials and requirements for repeated manual readings, respectively (14). The recent performance standards for antimicrobial susceptibility testing by CLSI mentioned zone diameter breakpoints for disk diffusion testing directly from positive blood culture broths for Enterobacterales with ampicillin, ceftriaxone, ceftazidime, aztreonam, meropenem, tobramycin, ciprofloxacin, and trimethoprim-sulfamethoxazole disks and for *Pseudomonas aeruginosa* with ceftazidime, meropenem, tobramycin, and ciprofloxacin disks only (12). Conversely, direct AST by established automated systems allows simultaneous identification of microbial pathogens and reporting of minimum inhibitory concentrations (MIC) of substantially more antimicrobials in a timely manner.

Therefore, we aimed to evaluate the CLSI-recommended direct AST methods and two other direct methods based on the disk diffusion method and automated AST in the present study. The study’s primary objective was to compare the performance of direct methods with the standard routine methods. The secondary objectives were to compare their performance between Enterobacterales and non-fermenters.

## MATERIALS AND METHODS

This study was conducted in the Clinical Microbiology Laboratories of the National Cancer Institute at the Jhajjar campus of All India Institute of Medical Sciences, New Delhi, India, between September 2022 and February 2023.

### Blood culture specimens

Blood for culture and susceptibility testing was obtained in BD BACTEC Plus Aerobic medium (Becton Dickinson, USA) or BD BACTEC Peds Plus medium (Becton Dickinson, USA) from hematological and solid organ malignancy patients admitted to the medical oncology, surgical oncology, radiation oncology, palliative care, and intensive care units of the study center. All bottles were immediately loaded in the BD BACTEC FX 40 blood culture system (Becton Dickinson, USA). Further processing was carried out when the system flagged a bottle positive.

Bottles were promptly removed from the system. Gram staining of the smear made from the flagged positive broth was performed.

### Selection criteria for specimens

Consecutive non-repetitive flagged positive blood culture broths showing only Gram-negative rods of similar morphotypes were included. In the case of multiple blood cultures from a single patient flagging positive, only one flagged positive blood culture broth per patient was included. Blood culture broths yielding polymicrobial growth in sub-culture on solid media were excluded.

### Standard inoculation protocol

Blood agar and MacConkey agar plates were inoculated with blood culture broths from the flagged positive blood culture bottles showing Gram-negative rods and incubated in a 5% CO_2_-rich environment at 37°C for 18–24 hours. Standardized bacterial inoculum (0.5 McFarland) was prepared from bacterial colonies isolated as pure culture on the solid media with the help of BD PhoenixSpec nephelometer (Becton Dickinson, USA). NMIC/ID panel (Becton Dickinson, USA) was inoculated with the standard bacterial suspension per the manufacturer’s instructions, and the panel was loaded into BD Phoenix M50 (Becton Dickinson, USA) for automated ID and AST of the bacterial isolate.

The Kirby-Bauer disk diffusion method was also performed using 10-cm diameter Mueller Hinton agar (MHA) plates (HiMedia Laboratories, India) and different antibiotic disks (HiMedia Laboratories, India) with the same standard bacterial inoculum and incubated at 35°C ± 2°C in ambient air for 16–18 hours. AST results were interpreted as per the performance standards for AST by CLSI (12).

### Direct inoculation protocols

Direct susceptibility testing was performed from flagged positive blood culture broths by three different protocols. The blood agar plates inoculated to obtain colonies for the standard inoculation protocol were checked to ensure pure growth before interpreting the AST using direct inoculation protocols.

#### Protocol A

Disk diffusion testing directly from the flagged positive blood culture bottles was performed using the CLSI-described method. Four to five drops of blood culture broths were dispensed onto MHA plates after thoroughly mixing by inverting the blood culture bottles 5–10 times. The blood culture broths were then spread on the entire surface of MHA using a sterile cotton swab (the procedure was repeated twice more by rotating the plate at 60°C to ensure even distribution of inoculum). The inoculated plate was kept lid ajar for 5 minutes before ampicillin (10 µg), ceftriaxone (30 µg), ceftazidime (30 µg), meropenem (10 µg), ciprofloxacin (5 µg), and trimethoprim-sulfamethoxazole (1.25/23.75 µg) disks (HiMedia Laboratories, India) were placed on the agar surface. Plates were incubated for 16–18 hours at 35°C ± 2°C in ambient air. Results are interpreted as per the zone diameter breakpoints for disk diffusion directly from flagged positive blood as per CLSI.

#### Protocol B

A 5-mL broth was aspirated from the flagged positive blood culture bottle using a sterile disposable syringe. The broth was then dispensed into a sterile BD Vacutainer SST II Advance tube and centrifuged at 2,000 × *g* for 15 minutes in the Neya 16R Centrifuge instrument (REMI, India). After centrifugation, the supernatant was discarded, and the bacterial pellets trapped in the gel layer were carefully harvested with StabiFlexLoop (HiMedia Laboratories, India) of 1.25-mm diameter. A 0.5 McFarland bacterial inoculum was prepared from the harvested bacterial pellets using BD PhoenixSpec nephelometer (Becton Dickinson, USA). NMIC/ID panel (Becton Dickinson, USA) was inoculated with the standard bacterial suspension per the manufacturer’s instructions. NMIC/ID panel is used for Gram-negative organisms only. Each panel contains a set of wells (five for each antibiotic) with varying concentrations of different antimicrobial agents. Additionally, there are 51 wells containing different identification substrates. The panel was loaded into the BD Phoenix M50 system (Becton Dickinson, USA) for automated ID and AST of the bacterial isolate. The system tracks the growth of microorganisms in all wells and displays identification and susceptibility results using the patterns of microbial growth.

#### Protocol C

Kirby-Bauer disk diffusion method was also performed using 10-cm diameter MHA plates (HiMedia Laboratories, India) and ampicillin (10 µg), amoxicillin-clavulanate (20/10 µg), piperacillin-tazobactam (100/10 µg), cefazolin (30 µg), ceftriaxone (30 µg), ceftazidime (30 µg), cefepime (30 µg), imipenem (10 µg), meropenem (10 µg), gentamicin (10 µg), amikacin (30 µg), ciprofloxacin (5 µg), levofloxacin (5 µg), and trimethoprim-sulfamethoxazole (1.25/23.75 µg) disks (HiMedia Laboratories, India) with the same bacterial inoculum from protocol B and incubated at 35°C ± 2°C in ambient air for 16–18 hours. AST results were interpreted as per the performance standards for AST by CLSI.

**Fig 1:**
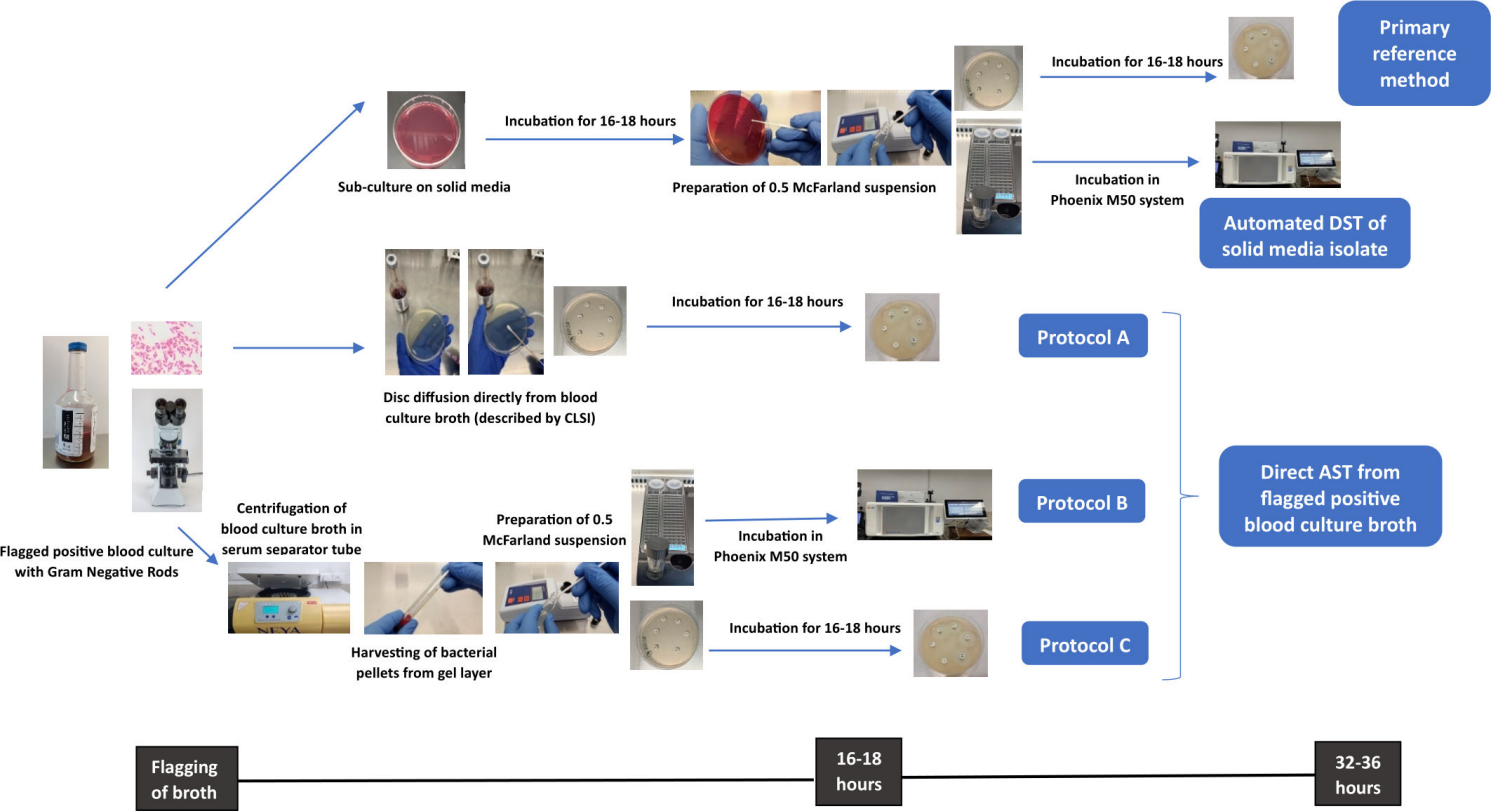
Workflow of standard inoculation and direct inoculation protocols.

The workflow of standard inoculation and direct inoculation protocols of AST is depicted in Fig. 1.

### Quality control (QC)

The disk diffusion methods used *E. coli* ATCC 25922 and *P. aeruginosa* ATCC 27853 strains for QC. *E. coli* ATCC 25922, *P. aeruginosa* ATCC 27853, *E. coli* ATCC 35218, and *Klebsiella pneumoniae* ATCC 700603 strains were used for QC in the AST by BD Phoenix M50 (Becton Dickinson, USA) system.

### Data analysis

The performance of the direct AST protocols was compared with the standard Kirby-Bauer disk diffusion method (primary reference method) with the solid media isolate and as expressed as categorical agreement (CA). Any disagreement between the direct AST protocols and the reference method was further categorized as minor error (mE), major error (ME), and very major error (VME) as per guidance on AST by the Food and Drug Administration (FDA) (15). mE was defined as an “intermediate” result by one method and either a “resistant” or “susceptible” result by the other method for a particular drug-bug combination. ME was defined as a “susceptible” result by the reference method but a “resistant” result by the direct AST method for a particular drug-bug combination. VME occurred when the reference method yielded a “resistant” result for a specific drug-bug combination, but the direct AST method yielded a “susceptible” result.

Additionally, we also calculated both the CA and essential agreement (EA; the difference in minimum inhibitory concentrations of a drug against a particular organism by two different methods were within ±1 twofold dilutions) for protocol B with respect to the automated AST by the BD Phoenix M50 system with the solid media isolate.

Minor error rates were calculated by dividing the number of minor errors by the total number of isolates tested. Major error rates were calculated by dividing the number of major errors by the number of susceptible isolates detected by the reference method, and very major error rates were calculated by dividing the number of very major errors by the number of resistant isolates detected by the reference method.

AST results of an organism against a particular drug with known intrinsic resistance were not considered for analyses.

### Statistical methods

Pearson’s χ^2^ test was used to compare the performance of direct inoculation protocols (protocols B and C) between Enterobacterales and non-fermenters. McNemar’s test was used to compare AST results between protocols B and C for Enterobacterales and non-fermenters separately. Mann-Whitney *U* test was performed for comparing durations to obtain AST results between Enterobacterales and non-fermenters by protocol B. *P*-value  <0.05 was considered statistically significant. All statistical calculations were performed in SPSS for Windows version 16 (SPSS, Inc., Chicago, IL, USA).

## RESULTS

A total of 1,640 blood culture broths were received for culture and susceptibility testing during the 6-month study period. A total of 297 specimens were flagged positive by the BD BACTEC FX 40 system. Gram-negative rods were identified by microscopy in 129 specimens. Finally, excluding duplicates, specimens yielding polymicrobial growth in solid media subculture, and invalid or no identification or AST results in the automated system, the AST results of 80 specimens were analyzed.

Majority of isolates were Enterobacterales (*Escherichia coli* 32, 40%; *Klebsiella pneumoniae* 14, 17.5%; *Salmonella* Typhi 6, 7.5%; *Serratia marcescens* 4, 5%; *Citrobacter freundii* 1, 1.25%; and *Enterobacter cloacae* 1; 1.25%) followed by non-fermenters (*Pseudomonas aeruginosa* 15, 18.75%; *Burkholderia cepacia* 3, 3.75%; *Acinetobacter baumannii* 2, 2.5%; *Stenotrophomonas maltophilia* 2, 2.5%). No discrepancy was observed in the ID of solid media isolates and direct ID from blood culture broth by the BD Phoenix M50 system.

After excluding known intrinsic resistance, 944 AST results (Enterobacterales 774 and non-fermenters 170) of all drug-bug combinations by protocols B and C were compared with the reference method (Table 1). On the other hand, only 373 AST results (Enterobacterales 328 and *Pseudomonas aeruginosa* 45) from protocol A were available for limited drug-bug combinations as per CLSI guidelines.

**TABLE 1:** Performance comparisons of direct AST protocols for different organisms^*a*^

Organisms	CA of direct AST protocols (based on the primary reference method), n/N (%)		
	Protocol A	Protocol B	Protocol C
Enterobacterales	319/328 (97.2)	744/774 (96.1)	745/774 (96.3)
*E. coli*	187/192 (97.4)	433/448 (96.7)	432/448 (96.4)
*K. pneumoniae*	66/70 (94.3)	170/182 (93.4)	173/182 (95.1)
*S*. Typhi	36/36 (100)	77/78 (98.7)	75/78 (96.2)
*S. marcescens*	20/20 (100)	44/44 (100)	44/44 (100)
*C. freundii*	5/5 (100)	9/11 (81.8)	10/11 (90.9)
*E. cloacae*	5/5 (100)	11/11 (100)	11/11 (100)
Non-fermenters		159/170 (93.5)	161/170 (94.7)
*P. aeruginosa*	45/45 (100%)	127/135 (94.1)	127/135 (94.1)
*B. cepacia*		8/9 (88.9)	8/9 (88.9)
*A. baumannii*		21/22 (95.5)	22/22 (100)
*S. maltophilia*		3/4 (75)	4/4 (100)
Total	364/373 (97.6%)	903/944 (95.7%)	906/944 (95.9%)

^*a*^ n = numerator; N = denominator.

### Performance of direct inoculation protocols for Enterobacterales

#### Based on the standard disk diffusion (primary reference method)

Protocols A, B, and C demonstrated 97.2%, 96.1%, and 96.3% categorical agreement among Enterobacterales, respectively. The categorical agreement, minor error, major error, and very major error rates of both protocols B and C for individual antibiotics against Enterobacterales were depicted in Table 2. Protocol B had 27 minor errors, 1 major error (for imipenem), and 2 very major errors (both for ceftazidime), and Protocol C had 26 minor errors, 2 major errors (one each for cefepime and gentamicin), and 1 very major error (for trimethoprim-sulfamethoxazole). Overall, minor error, major error, and very major error rates of protocol B were 3.5%, 0.36%, and 0.43%, respectively, whereas minor error, major error, and very major error rates of protocol C were 3.4%, 0.72%, and 0.21%, respectively.

**TABLE 2:** Categorical agreement and error rates in direct AST protocols B and C (based on the primary reference method) for individual drugs against Enterobacterales^*a*^

Drugs	Protocol B				Protocol C			
	CA, n/N (%)	mE rate, n/N (%)	ME rate, n/N (%)	VME rate, n/N (%)	CA, n/N (%)	mE rate, n/N (%)	ME rate, n/N (%)	VME rate, n/N (%)
Ampicillin	38/38 (100)	0/38	0/10	0/28	37/38 (97.4)	1/38 (2.6)	0/10	0/28
Amoxicillin-clavulanate	50/52 (96.2)	2/52 (3.8)	0/10	0/35	48/52 (92.3)	4/52 (7.7)	0/10	0/35
Piperacillin-tazobactam	54/58 (93.1)	4/58 (6.9)	0/22	0/29	56/58 (96.6)	2/58 (3.4)	0/22	0/29
Cefazolin	50/52 (96.2)	2/52 (3.8)	0/5	0/47	52/52 (100)	0/52	0/5	0/47
Ceftriaxone	57/58 (98.3)	1/58 (1.7)	0/12	0/45	57/58 (98.3)	1/58 (1.7)	0/12	0/45
Ceftazidime	53/58 (91.4)	3/58 (5.2)	0/11	2/44 (4.5)	54/58 (93.1)	4/58 (6.9)	0/11	0/44
Cefepime	55/58 (94.8)	3/58 (5.2)	0/18	0/38	56/58 (96.6)	1/58 (1.7)	1/18 (5.6)	0/38
Imipenem	53/58 (91.4)	4/58 (6.9)	1/34 (2.9)	0/23	54/58 (93.1)	4/58 (6.9)	0/34	0/23
Meropenem	57/58 (98.3)	1/58 (1.7)	0/33	0/23	56/58 (96.6)	2/58 (3.4)	0/33	0/23
Gentamicin	57/58 (98.3)	1/58 (1.7)	0/39	0/19	56/58 (96.6)	1/58 (1.7)	1/39 (2.6)	0/19
Amikacin	57/58 (98.3)	1/58 (1.7)	0/39	0/17	56/58 (96.6)	2/58 (3.4)	0/39	0/17
Ciprofloxacin	56/58 (96.6)	2/58 (3.4)	0/8	0/47	55/58 (94.8)	3/58 (5.2)	0/8	0/47
Levofloxacin	51/52 (98.1)	1/52 (1.9)	0/10	0/42	51/52 (98.1)	1/52 (1.9)	0/10	0/42
Trimethoprim-sulfamethoxazole	56/58 (96.6)	2/58 (3.4)	0/28	0/30	57/58 (98.3)	0/58	0/28	1/30 (3.3)
Total	744/774 (96.1)	27/774 (3.5)	1/279 (0.36)	2/467 (0.43)	745/774 (96.3)	26/774 (3.4)	2/279 (0.72)	1/467 (0.21)

^*a*^ n = numerator; N = denominator; CA = categorical agreement; mE = minor error; ME = major error; VME = very major error.

#### Based on the automated AST of solid media subcultures

The overall categorical agreement and essential agreement between protocol B and automated AST (solid media isolates) were 99.1% and 99.5%, respectively. The essential agreement was 100% for most of the antibiotics except imipenem (against one *E. coli* isolate), ceftazidime (against one *E. coli* isolate), ciprofloxacin (against one *E. coli* isolate), and trimethoprim-sulfamethoxazole (against one *E. coli* isolate).

The mean duration for obtaining the AST results of Enterobacterales as per protocol B after loading on the automated system was 13.73 ± 2.2 (SD) hours.

### Performance of direct inoculation protocols for non-fermenters

#### Based on the standard disk diffusion (primary reference method)

Protocol A had 100% categorical agreement in *P. aeruginosa*. Categorical agreement of protocols B and C among non-fermenters were 93.5% and 94.7%, respectively. Minor error, major error, and very major error rates of both protocols B and C for individual antibiotics among non-fermenters were depicted in Table 3. Protocol B had 11 minor errors but no major errors or very major errors, and protocol C had seven minor errors and 2 major errors (one each for imipenem and amikacin) but no very major errors. Protocol B had a minor error rate of 6.5%, and protocol C had a minor error rate of 4.1% and an ME rate of 1.9%.

**TABLE 3:** Categorical agreement and error rates in direct AST protocols B and C (based on the primary reference method) for individual drugs against non-fermenters^*a*^

Drugs	Protocol B				Protocol C			
	CA, n/N (%)	mE rate, n/N (%)	ME rate, n/N (%)	VME rate, n/N (%)	CA, n/N (%)	mE rate, n/N (%)	ME rate, n/N (%)	VME rate, n/N (%)
Ceftriaxone	2/2 (100)	0/2	0/0	0/2	2/2 (100)	0/2	0/0	0/2
Ceftazidime	18/20 (90)	2/20 (10)	0/12	0/5	18/20 (90)	2/20 (10)	0/12	0/5
Cefepime	15/17 (88.2)	2/17 (11.8)	0/12	0/5	16/17 (94.1)	1/17 (5.9)	0/12	0/5
Piperacillin-tazobactam	16/17 (94.1)	1/17 (5.9)	0/13	0/4	15/17 (88.2)	2/17 (11.8)	0/13	0/4
Imipenem	16/17 (94.1)	1/17 (5.9)	0/7	0/10	16/17 (94.1)	0/17	1/7 (14.3)	0/10
Meropenem	19/20 (95)	1/20 (5)	0/6	0/14	19/20 (95)	1/20 (5)	0/6	0/14
Gentamicin	16/17 (94.1)	1/17 (5.9)	0/12	0/4	17/17 (100)	0/17	0/12	0/4
Amikacin	17/17 (100)	0/17	0/13	0/4	16/17 (94.1)	0/17	1/13 (7.7)	0/4
Ciprofloxacin	16/17 (94.1)	1/17 (5.9)	0/12	0/4	17/17 (100)	0/17	0/12	0/4
Levofloxacin	17/19 (89.5)	2/19 (10.5)	0/12	0/3	18/19 (94.7)	1/19 (5.3)	0/12	0/3
Trimethoprim-sulfamethoxazole	7/7 (100)	0/7	0/6	0/1	7/7 (100)	0/7	0/6	0/1
Total	159/170 (93.5)	11/170 (6.5)	0/105	0/56	161/170 (94.7)	7/170 (4.1)	2/105 (1.9)	0/56

^*a*^ n = numerator; N = denominator; CA = categorical agreement; mE = minor error; ME = major error; VME = very major error.

#### Based on the automated AST of solid media subcultures

The overall categorical agreement and essential agreement between protocol B and automated AST (solid media isolates) were 98.2% and 97.6%, respectively. The essential agreement was 100% for most of the antibiotics except imipenem (against one *P. aeruginosa* isolate), ceftazidime (against one each of *P. aeruginosa* and *B. cepacia* isolates), and cefepime (against one *P. aeruginosa* isolate).

After loading on the automated system, the mean duration for obtaining the AST results of non-fermenters as per protocol B was 15.27 ± 1.83 (SD) hours.

### Comparative analysis

Based on the standard disk diffusion (primary reference method), the performance of protocols B and C (the overall CA) was compared individually between Enterobacterales and non-fermenters. The performance of protocol B did not significantly differ between Enterobacterales and non-fermenters (Pearson’s χ^2^ *P*-value of 0.13). Similarly, the performance of protocol C did not significantly differ between Enterobacterales and non-fermenters (Pearson’s χ^2^ *P*-value of 0.35).

On the other hand, in the sub-group analysis (Enterobacterales and non-fermenters sub-groups), the performance (the overall CA) between protocols B and C was compared. Statistically significant differences in performance between protocols B and C were observed neither for Enterobacterales (McNemar’s test *P*-value of 1) nor for non-fermenters (McNemar’s test *P*-value of 0.68).

The difference in duration for obtaining the AST results by protocol B between Enterobacterales and non-fermenters was not statistically significant (*P* = 0.098).

## DISCUSSION

The present study focused on evaluating the performance of three direct inoculation protocols for AST against Gram-negative rods directly from blood culture bottles. Previous studies have demonstrated variable performance of different AST protocols from flagged positive blood culture systems using different automated systems (16–19). Some studies also compared the performance of two different automated systems for performing direct susceptibility from flagged positive blood culture bottles (5). Few more studies also stressed the possibility of performing a disk diffusion method using 0.5 McFarland inoculum prepared directly from blood culture broths for resource-limited settings lacking automated AST systems (16, 20, 21). In our study, we not only compared an automated method and a disk diffusion method for direct susceptibility testing but also performed and assessed the performance of an already standardized direct blood culture disk diffusion protocol as prescribed by the CLSI.

In the validation study of the CLSI direct blood culture disk diffusion protocol, the overall categorical agreement for Gram-negative rods varied from 87.8% (BacT/Alert) to 92.2% (VersaTREK) for different automated blood culture systems (20). In our study, the overall categorical agreement (based on the primary reference method) of the CLSI direct protocol (protocol A) was 97.6% (97.2% for Enterobacterales and 100% for *P. aeruginosa*), which was better than the categorical agreement (88.4%) reported in the validation study from the same BACTEC blood culture broths (22). Although the CLSI method performed extraordinarily compared to the routine disk diffusion method, it allowed testing against a handful of antibiotics for Enterobacterales and *P. aeruginosa* only (12).

On the other hand, both protocols B and C allowed testing against a significantly increased number of antibiotics using the standard CLSI breakpoints (12). We found that the overall categorical agreements (based on the primary reference method) of protocols B and C for Enterobacterales were 96.1% (3.5% minor error, 0.36% major error, and 0.43% very major error rates) and 96.3% (3.4% minor error, 0.72% major error, and 0.21% very major error rates), respectively, whereas those of protocols B and C for non-fermenters were 93.5% (6.5% minor error rate) and 94.7% (4.1% minor error and 1.9% major error rates), respectively. All these results for both Enterobacterales and non-fermenters groups were acceptable per the FDA guidelines for AST systems (15). Gherardi et al. also reported that the categorical agreement of the direct AST method (using the Phoenix system) for Gram-negative rods with the standard method was >95% in their study (5). On the other hand, Kumar et al. in their study on direct susceptibility by Kirby-Bauer disk diffusion method using standard inoculum prepared from centrifuged bacterial pellets reported 98.95% categorical agreement (0.42% minor error, 0.42% major error, and 0.21% very major error rates) and 98.21% categorical agreement (0.60% minor error and 1.19% major error rates) in the Enterobacterales and non-fermenters groups, respectively (20). Kavipriya et al. reported comparatively better categorical agreement for non-fermenters (97.6%) than Enterobacterales (97%) using the Vitek-2 system for direct AST from blood culture broths (18). Conversely, Gomez et al. reported much better categorical agreement for Enterobacterales (96.67%) compared to non-fermenters (92.3%) using the same system (17). We found that the performance of direct AST protocols (B and C) between Enterobacterales and non-fermenters was not statistically different. Moreover, a significant difference between the performance of protocols B and C was also found neither among Enterobacterales nor among non-fermenters.

In our study, the categorical agreement and essential agreement between protocol B and automated AST of solid media subcultures were 99.1% (767/774) and 99.5% (770/774), respectively, among Enterobacterales. Among non-fermenters, the above methods’ categorical agreement and essential agreement were 98.2% (167/170) and 97.6% (166/170), respectively. Gherardi et al. reported that the categorical agreement between automated AST from solid media subculture and directly from blood culture broths in Gram-negative rods (using the Phoenix system for both) was 99%, which was similar to the overall categorical agreement (98.9%, 934/944) found between above methods in our study (5).

Direct AST methods performed with blood culture broths allow early administration of optimal therapy and improve antimicrobial stewardship for patients with bloodstream infections (9). An effective direct method from flagged positive blood culture broths can reduce the turnaround time of AST results by 24 hours, which is crucial to clinicians for deciding on or modifying specific antimicrobial treatments (18, 20). A previous study reported that the mean duration for AST results for Gram-negative rods by direct method with the Phoenix system was 11.76 ± 2.72 (SD) hours (5). We found that the mean durations for obtaining the AST results of Enterobacterales and non-fermenters as per protocol B were 13.73 ± 2.2 (SD) hours and 15.27 ± 1.83 (SD) hours, respectively, and were not statistically different.

Both direct inoculation protocols (B and C) demonstrated excellent performance with respect to the standard disk diffusion (primary reference method) for susceptibility testing from blood culture broths. The added advantage of protocol B (based on the automated system) was the simultaneous identification of the isolates along with AST, whereas a separate identification method must be incorporated with protocols A and C, which provided only AST results. Gherardi et al. found only a 3.3% (3/91) mismatch between automated identification from solid media subculture and automated identification directly from blood culture broths for Gram-negative rods (both by the Phoenix system) (5). Interestingly, no discrepancy was found between identification by the automated method of solid media subcultures and protocol B in the present study.

Polymicrobial cultures and low inoculum size are primary barriers to successfully implementing direct AST protocols from blood cultures (5, 22). We excluded samples based on variable morphology and staining properties of bacteria by Gram staining of smears prepared from broths and polymicrobial growth in solid media subcultures. Thus, a follow-up subculture on solid media alongside the direct AST protocols should be performed to ensure appropriate interpretation of AST results and identification. The issue of low inoculum size was well addressed in our study, where we ensured the preparation of 0.5 McFarland standard suspension with meticulously harvested bacterial cell pellets trapped in the gel layer of the serum separator vials after centrifugation. Similar bacterial cell pellet harvesting protocols were applied by other authors, describing accurate direct AST results from blood cultures using a different automated system (23).

Our study had a few limitations. First, it was a single-center study with only 80 specimens. Although similar studies included the same or even fewer isolates to validate their protocols, multicentric studies with more specimens could have increased the validity of the findings (5, 14, 24). Second, the present study’s findings are restricted to Gram-negative isolates only. Few studies reported poor performance of direct AST protocols for Gram-positive cocci (22, 25). Our future endeavor would be to evaluate protocols B and C for Gram-positive organisms. Third, this study lacked definitive identification of bacterial isolates by sequencing or Matrix Assisted Laser Desorption Ionization-Time of Flight (MALDI-TOF) and solely relied on the ID by automated ID-AST system.

### Conclusion

All three direct AST protocols demonstrated acceptable performance with excellent categorical agreements with primary reference method as per established guidelines. Performance of protocols B and C between Enterobacterales and non-fermenters was not statistically different. Similarly, the performance of both protocols B and C was statistically similar. Both categorical and essential agreements between protocol B and automated AST of solid media subcultures were excellent.
